# Perceptions and Utilization of Online Peer Support Among Informal Dementia Caregivers: Survey Study

**DOI:** 10.2196/55169

**Published:** 2024-05-31

**Authors:** Zhijun Yin, Lauren Stratton, Qingyuan Song, Congning Ni, Lijun Song, Patricia Commiskey, Qingxia Chen, Monica Moreno, Sam Fazio, Bradley Malin

**Affiliations:** 1 Department of Biomedical Informatics, Vanderbilt University Medical Center, Nashville, TN, United States; 2 Department of Electrical Engineering and Computer Science, Vanderbilt University, Nashville, TN, United States; 3 Psychosocial Research and Program Evaluation, Alzheimer's Association, Chicago, IL, United States; 4 Department of Sociology, Vanderbilt University, Nashville, TN, United States; 5 Department of Neurology, Vanderbilt University Medical Center, Nashville, TN, United States; 6 Department of Biostatistics, Vanderbilt University Medical Center, Nashville, TN, United States; 7 Care and Support, Alzheimer’s Association, Chicago, IL, United States; 8 Quality Care and Psychosocial Research, Alzheimer’s Association, Chicago, IL, United States

**Keywords:** informal dementia caregiver, online health community, social support, survey, online peer support, caregiving challenges

## Abstract

**Background:**

Informal dementia caregivers are those who care for a person living with dementia and do not receive payment (eg, family members, friends, or other unpaid caregivers). These informal caregivers are subject to substantial mental, physical, and financial burdens. Online communities enable these caregivers to exchange caregiving strategies and communicate experiences with other caregivers whom they generally do not know in real life. Research has demonstrated the benefits of peer support in online communities, but this research is limited, focusing merely on caregivers who are already online community users.

**Objective:**

We aimed to investigate the perceptions and utilization of online peer support through a survey.

**Methods:**

Following the Andersen and Newman Framework of Health Services Utilization and using REDCap (Research Electronic Data Capture), we designed and administered a survey to investigate the perceptions and utilization of online peer support among informal dementia caregivers. Specifically, we collected types of information that influence whether an informal dementia caregiver accesses online peer support: predisposing factors, which refer to the sociocultural characteristics of caregivers, relationships between caregivers and people living with dementia, and belief in the value of online peer support; enabling factors, which refer to the logistic aspects of accessing online peer support (eg, eHealth literacy and access to high-speed internet); and need factors, which are the most immediate causes of seeking online peer support. We also collected data on caregivers’ experiences with accessing online communities. We distributed the survey link on November 14, 2022, within two online locations: the Alzheimer’s Association website (as an advertisement) and ALZConnected (an online community organized by the Alzheimer’s Association). We collected all responses on February 23, 2023, and conducted a regression analysis to identifyn factors that were associated with accessing online peer support.

**Results:**

We collected responses from 172 dementia caregivers. Of these participants, 140 (81.4%) completed the entire survey. These caregivers were aged 19 to 87 (mean 54, SD 13.5) years, and a majority were female (123/140, 87.9%) and White (126/140, 90%). Our findings show that the behavior of accessing any online community was significantly associated with participants’ belief in the value of online peer support (*P*=.006). Moreover, of the 40 non–online community caregivers, 33 (83%) had a belief score above 24—the score that was assigned when a neutral option was selected for each belief question. The most common reasons for not accessing any online community were having no time to do so (14/140, 10%) and having insufficient online information–searching skills (9/140, 6.4%).

**Conclusions:**

Our findings suggest that online peer support is valuable, but practical strategies are needed to assist informal dementia caregivers who have limited time or online information–searching skills.

## Introduction

Alzheimer disease—the most common cause of dementia—is a brain disorder that affects the thinking, comprehension, and learning capacity of more than 6 million Americans and is the seventh leading cause of death in the United States [1]. An estimated 80% of people living with Alzheimer disease or related dementia are cared for by unpaid informal caregivers (eg, family members, friends, or other unpaid caregivers) [2]. In 2021, over 11 million informal dementia caregivers provided 16 billion hours of care to people living with dementia [1]. Although this care was valued at nearly US $271.6 billion, it imposed substantial physical, financial, and mental burdens on these informal caregivers [3]. Additionally, 30% of informal dementia caregivers are aged ≥65 years [1]. They are likely to experience reduced social engagement due to caring for people living with dementia, which increases their risk of developing Alzheimer disease or some other dementia [4-6] and their risk of early death [7,8]. To ensure sufficient support for both informal caregivers and care recipients, it is essential for society to develop effective support mechanisms for the needs of informal dementia caregivers [9].

Research on how to best support informal dementia caregivers has focused primarily on assistance from credentialed professionals [10-12]. This type of assistance can improve a caregiver’s emotional well-being and caring skills, but maintaining this assistance over time can be difficult to achieve on a large scale. This is due, in part, to an insufficiently sized workforce, limited financial support [13,14], the stigma of asking for help, and difficulties encountered when leaving individuals with dementia [15,16]. In addition, if they lack a shared experience, it may be difficult for health care professionals or other family members to respond to the specific needs of informal caregivers. The perception that “they simply do not understand” [17] can contribute to feelings of loneliness [18], which were found to be negatively associated with the health and well-being of these caregivers [19].

The integration of the internet into daily life has enabled many people, including informal caregivers, to discuss health-related topics on online social media platforms [20,21]. For example, ALZConnected [22], which is organized by the Alzheimer’s Association, is the largest online community for people living with Alzheimer disease or related dementia and their caregivers in North America. ALZConnected has accumulated tens of thousands of online community users to discuss a broad range of topics regarding dementia caregiving and disease management [23]. Through online communities, informal dementia caregivers seek support and are willing to share experiences and practical information that they believe will assist other caregivers [24]. A study that analyzed an Alzheimer caregiver group on Facebook found that online peer social support had decreased the caregivers’ burdens while increasing their emotional and informational well-being [3]. Similarly, a survey found that increased online activity among caregivers was associated with lower levels of depression and loneliness [25]. In addition, many online communities provide added benefits, such as anonymity, asynchronous participation, and connection to numerous caregivers without physical location and time constraints [26], which provide cost-effective and convenient ways for informal dementia caregivers to gain support and access resources.

Based on internet utilization, informal dementia caregivers can be categorized as (1) *non-internet caregivers*, who never use the internet; (2) *non–online community caregivers*, who use the internet but do not participate in online communities; and (3) *online community caregivers*, who both use the internet and participate in online communities. Current social media–based dementia caregiving research primarily focuses on online community caregivers [25-27]. Although improving the online experiences of these caregivers is important [28], understanding how non–online community caregivers perceive the value of online peer support is significant as well. This would inform the development of interventions for non–online community caregivers to use and benefit from online peer support, thereby helping to mitigate the potential digital divide and decreasing existing health disparities in accessing online peer support [29].

The primary objective of this research was to gain insight into the perceptions and utilization of online community support among informal dementia caregivers, specifically non–online community caregivers and online community caregivers. To do so, we designed and administered a survey based on the Andersen and Newman Framework of Health Services Utilization (ANFHSU) [30]. The ANFHSU is a classical model for identifying and describing the factors that may affect a person’s access to and utilization of health services. Within this framework, we can analyze the various factors gathered through a survey questionnaire to determine an informal dementia caregiver’s access to or utilization of online peer support. Our findings suggest that online peer support is valuable, but practical strategies are needed to assist caregivers with limited time or online information–searching skills. This investigation marks the initial step toward addressing a long-term research objective, aiming to comprehensively elucidate the intricate mechanisms underlying online social support in dementia caregiving.

## Methods

### Online Peer Support

We begin this section with the definition of *online peer support*. Although peer support groups can be organized in an online format (eg, via Zoom meetings), the online peer support in this paper refers to the communications among informal dementia caregivers who may not know each other in real life but connect in online communities, forums, or websites (eg, Twitter, Facebook, Reddit, or ALZConnected). More broadly, since reading online caregiving discussions from other caregivers can serve as a way to obtain information and resources or learn caregiving skills, we also treated reading online posts as an online peer support–seeking behavior.

### Ethical Considerations

This study was reviewed and deemed as non-human subjects research by the Vanderbilt University Medical Center Institutional Review Board (IRB 221732). Each survey participant was asked to confirm that they were unpaid informal dementia caregivers and that they were voluntarily participating in this survey in an information sheet. The sheet also included the definitions of *informal Alzheimer disease and related dementia caregivers* and *online communities*; the research purpose; a notice that the data would be collected, processed, and used; the methods applied to protect participant privacy; and a contact number for the Institutional Review Board.

### Questionnaire Design

**Figure 1.**
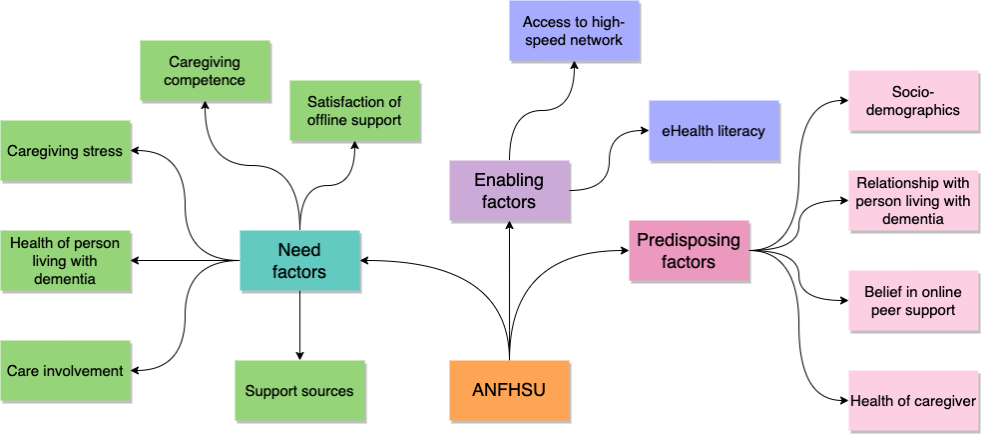
The predisposing, enabling, and need factors in the ANFHSU. ANFHSU: Andersen and Newman Framework of Health Services Utilization.

Social support is valuable in improving personal health [31]. In this study, we designed the survey based on the ANFHSU, whereby an informal dementia caregiver’s access to or utilization of online peer support was considered to be a function of 3 characteristics (Figure 1).

*Predisposing factors* were the sociocultural characteristics of individuals or other factors that existed before these individuals became dementia caregivers. Specifically, we included the following predisposing factors:

- Sociodemographics: these included age, biological sex, education level, race, ethnicity, occupational status, and income; we also inquired about the number of children being cared for (if applicable), acknowledging the significant challenges in life balance experienced by caregivers in the “sandwich generation” [23].
- Relationship between the caregiver and a person living with dementia: this was included because caregivers who have different relationships with a person living with dementia face different caregiving challenges and burdens.

- Belief in the value of online peer support: we asked the following seven 7-point Likert scale (ranging from “Strongly disagree” to “Strongly agree”) questions: “Do you believe that reading online discussions from, or directly writing posts to discuss with, other caregivers whom you do not know in the real world will help a) find caregiving resources that you need, b) increase caregiving knowledge, c) increase understanding of the disease and patient, d) improve caregiving skills, e) increase confidence in caregiving, f) reduce caregiving stress or g) reduce the feeling of loneliness as a caregiver?”
- Health of the caregiver: we collected data on this factor through the following 7-point Likert scale (ranging from “Extremely disagree” to “Extremely agree”) item: “I feel healthy and do not have any major diseases that affect my daily life.”

*Enabling factors* referred to the logistic aspects of accessing online peer support. The following enabling factors were included:

- Access to a high-speed network, which is an important contributor to the digital divide [32].
- eHealth literacy: this was measured through the eHealth Literacy Scale (eHEALS) [33] to evaluate if a caregiver could process online health-related information.

*Need factors* referred to the most immediate causes of seeking online peer support. The following need factors were included:

- Health of the person living with dementia: for simplicity, caregivers reported the dementia stage for people living with dementia as “early,” “middle,” or “late.”
- Care involvement: this was measured through two questions (“How long have you been taking care of the PLWD (caregiving duration)?” and “How frequently do you care for the PLWD per week (caregiving workload)?”), and this design was based on the facts that dementia caregiving is a long-term dynamic process and that the weekly caregiving workload would affect the need for online peer support.
- Caregiving challenges: data on these were obtained via an open-ended question that requested caregivers to indicate the most challenging issues that they have faced when caring for a person living with dementia.
- Offline support sources: we also asked caregivers to describe (in a textbox) where to obtain support in the real world to handle the aforementioned challenges.
- Satisfaction with any support received in offline environments (7-point Likert scale).
- Caregiving stress: improving psychological well-being is a key focus of many interventions. The intuition is that higher levels of stress in caregivers often drive them to seek support. We measured this factor by using the Zarit Burden Interview (12-item) scale [34].
- Caregiver competence: this factor referred to one’s self-evaluation of their capacity to care for a person living with dementia and was measured by using the CARERS (Coaching, Advocacy, Respite, Education, Relationship, and Simulation) Interview (4-item) scale [35].

The rationale of the design of the survey was that if an informal dementia caregiver finds offline support to be insufficient in solving their caregiving challenges, they may turn to online environments for peer support. In addition to the ANFHSU factors, we surveyed each participant’s experiences with using online peer support in the past 3 months (ie, their behavior). For the survey participants who were already online community users, we asked the following questions: (1) “Which online platforms have you visited in the past three months?” (2) “How frequently did you visit those online communities?” (3) “Will you intend to revisit these online communities in the next three months (intention)?” (4) “If the answer to question 3) is yes, what are your motivations for revisiting these online communities?” For participants who were not online community users, we asked them an open-ended question regarding why they did not seek online peer support in the past 3 months.

### Implementation and Dissemination

We implemented the survey questionnaire in REDCap (Research Electronic Data Capture; Vanderbilt University) [36]—a secure web application for building and managing online surveys. We distributed the survey link within 2 online locations. The first was ALZConnected, where we posted the survey link at the top of the thread board in the community’s two major caregiver forums: (1) *Caregivers Forum* and (2) *Spouse or Partner Caregiver Forum*. The second was the Alzheimer’s Association website [37], where an advertisement for this survey was created to allow any person who visited the website to have a chance to access the survey link. Given that this website is a popular source of information for patients with dementia and dementia caregivers, we expected to obtain survey responses from caregivers who were not part of online communities.

### Analysis

We performed three types of analyses. First, we summarized the answers to the multiple-choice questions by illustrating the respondent distribution for each choice. This was done to paint a broad picture of who the responding caregivers were and their perceptions and utilization of online peer support.

Second, we summarized the answers to the open-ended questions through manual annotation. This was not a trivial task because the responses were written as free text, and there were no predefined categories before the annotation. To address these issues, two authors (CN and QS) read the responses and annotated the categories for each open-ended question independently. Next, both annotators compared and discussed their summarized categories to create the final categories, including category names and definitions. Both annotators then independently modified their annotations with the agreed upon categories. For each response, we adopted a conservative approach and only reported the interactions of the categories summarized by the two annotators.

Finally, we fitted a logistic regression model (R v4.2.2; R Foundation for Statistical Computing) to analyze how the proposed ANFHSU factors were associated with using online peer support. Due to the small sample size, we converted some categorical variables into binary ones. Specifically, we encoded *gender* as “female/non-female,” *race* as “white/non-white,” *education level* as “4-year college degree or above/below 4-year college degree,” *access to high-speed network* as “yes/no,” *relationship* as “spouse/non-spouse,” and all the other categorical variables (eg, *annual income*, *dementia stage*, *caregiving duration*, and *caregiving workload*) as ordinal variables (eg, numerical values with equal distance). A 2-sided *P* value of <.05 was considered to be statistically significant.

## Results

### Survey Overview

We distributed the survey link on November 14, 2022, leaving it open for over 3 months until February 23, 2023. During this time period, we collected responses from 172 dementia caregivers. Of these participants, 140 (81.4%) completed the entire survey.

### Characteristics of Caregivers and People Living With Dementia

Table 1 summarizes the sociodemographic and caregiving characteristics of the 140 caregivers who completed the survey. These caregivers were aged 19 to 87 (mean 54, SD 13.5) years, and a majority were female (123/140, 87.9%) and White (126/140, 90%). Over 60% (89/140, 63.6%) of these caregivers had a 4-year college degree or higher, whereas 36.4% (51/140) had a below–4-year college education level. A majority of the caregivers were the adult children of the people living with dementia (71/140, 50.7%), followed by spouses or partners (41/140, 29.3%) and other relatives (19/140, 13.6%). Only 40.7% (57/140) of these caregivers were employed full-time, and 28.6% (40/140) took care of the person living with dementia and at least one child simultaneously. Further, 17.9% (25/140) and 32.1% (45/140) of the respondents reported taking care of the person living with dementia for <1 year and >4 years, respectively, and 66.4% (93/140) provided daily care. The caregivers’ annual income was approximately uniformly distributed across incomes of less than US $25,000 to incomes ranging between US $100,000 and US $149,999. Only 7.9% (11/140) of these caregivers earned more than US $149,999.

Table 2 presents the characteristics of the people living with dementia reported by 140 survey participants. The ages of the people living with dementia ranged from 46 to 97 (mean 76, SD 9.5) years, and 65% (91/140) were female. Over 60% (86/140, 61.4%) of the people living with dementia were at the middle stage of dementia, 21.4% (30/140) were at the late stage, and 17.1% (24/140) were at the early stage.

**Table 1.** Summary of the sociodemographics and caregiving characteristics of the 140 survey caregiver participants.

Characteristic		Caregivers (N=140)
Age (y), mean (SD; range)		54 (13.5; 19-87)
**Gender, n (%)**		
	Female	123 (87.9)
	Male	16 (11.4)
	Undifferentiated	1 (0.7)
**Race, n (%)**		
	White	126 (90)
	Asian	8 (5.7)
	Black or African American	5 (3.6)
	Unknown	1 (0.7)
**Ethnicity, n (%)**		
	Not Hispanic or Latino	130 (92.9)
	Hispanic or Latino	10 (7.1)
**Education level, n (%)**		
	Above a 4-y college degree	57 (40.7)
	4-y college graduate	32 (22.9)
	Some college or 2-y degree	40 (28.6)
	High school or General Educational Development	11 (7.9)
**Employment status, n (%)**		
	Full time	57 (40.7)
	Retired	40 (28.6)
	Part time	22 (15.7)
	Unemployed	21 (15)
**Relationship, n (%)**		
	Adult child	71 (50.7)
	Spouse or partner	41 (29.3)
	Other relative	19 (13.6)
	Grandchild	6 (4.3)
	Neighbor	1 (0.7)
	Friend	1 (0.7)
	Other	1 (0.7)
**Number of children cared for, n (%)**		
	1	15 (10.7)
	2	20 (14.3)
	3	2 (1.4)
	>3	3 (2.1)
	Does not apply	100 (71.4)
**Caregiving duration, n (%)**		
	<6 mo	12 (8.6)
	6-12 mo	13 (9.3)
	1-2 y	35 (25)
	2-4 y	35 (25)
	>4 y	45 (32.1)
**Caregiving workload, n (%)**		
	<1 time per week	6 (4.3)
	1-2 times per week	21 (15)
	3-6 times per week	20 (14.3)
	Daily	93 (66.4)
**Annual income (US $), n (%)**		
	<25,000	24 (17.1)
	25,000-49,999	30 (21.4)
	50,000-74,999	26 (18.6)
	75,000-99,999	21 (15)
	100,000-149,999	28 (20)
	≥150,000 or more	11 (7.9)

**Table 2.** Characteristics of the people living with dementia reported by survey participants.

Characteristic		People living with dementia (N=140)
Age (y), mean (SD; range)		76 (9.5; 46-97)
**Gender, n (%)**		
	Female	91 (65)
	Male	49 (35)
**Dementia stage, n (%)**		
	Early stage	24 (17.1)
	Middle stage	86 (61.4)
	Late stage	30 (21.4)

### Caregiving Challenges and Support Sources

Table 3 summarizes reported caregiving challenges and the sources where caregivers sought support. The main caregiving challenges included dealing with the memory issues of a person living with dementia (40/140, 28.6%), supporting a person living with dementia in their daily life (such as showering and transportation; 30/140, 21.4%), and maintaining a balanced life (24/140, 17.1%). It should be noted that life balancing included the ability to (1) balance taking care of one’s children and a person living with dementia, (2) balance work and caregiving, and (3) balance one’s social life and caregiving. Dealing with the emotional fluctuations of the person living with dementia (21/140, 15%) and dealing with financial issues (14/140, 10%) were also major caregiving challenges. Some caregivers expressed concerns about the dementia treatment (11/140, 7.9%) for people living with dementia and their own mental health issues (10/140, 7.1%).

**Table 3.** Summary of caregiving challenges and specific support sources.^a^

		Caregivers (N=140), n (%)
**Challenge**		
	Legal issue	1 (0.7)
	Physical health	1 (0.7)
	Other health issue	2 (1.4)
	Mental health (caregiver)	10 (7.1)
	Treatment concern	11 (7.9)
	Family conflict	13 (9.3)
	Financial issue	14 (10)
	Emotion change	21 (15)
	Life balancing	24 (17.1)
	Daily caregiving	30 (21.4)
	Memory issue	40 (28.6)
**Support source**		
	Self-learning (book)	4 (2.9)
	Dementia caregivers (offline)	7 (5)
	Self-learning (online search)	8 (5.7)
	Local support group	23 (16.4)
	Friend	35 (25)
	Dementia caregivers (online community)	35 (25)
	Health care provider	38 (27.1)
	Family member	66 (47.1)

a Caregivers could report multiple challenges and support sources, which is why the total of the percentages for each section exceed 100%.

### Satisfaction With Support and Network Access

Table 4 depicts the distribution of caregivers by satisfaction with the support they sought and by their access to a high-speed network. Of the 140 caregivers, 69 (49.3%) reported an “agree” or above response (which included “Slightly agree,” “Quite agree,” and “Extremely agree”) for their satisfaction with the sought support. Among the 56 (40%) caregivers who reported negative experiences, 12 (8.6%) reported “Extremely disagree,” indicating the challenging situations faced by these caregivers. Only 6 caregivers reported that they could not access a high-speed network.

**Table 4.** Distribution of caregivers by satisfaction with support that was sought and by access to a high-speed network.

Items and responses		Caregivers, n
**Satisfaction with the sought support**		
	Extremely disagree	12
	Quite disagree	29
	Slightly disagree	15
	Neither	15
	Slightly agree	37
	Quite agree	31
	Extremely agree	1
**Access to a high-speed network**		
	Extremely disagree	1
	Quite disagree	2
	Slightly disagree	3
	Slightly agree	11
	Quite agree	38
	Extremely agree	85

### Measures of Caregiving Stress and Competence, eHEALS, and Belief

**Figure 2.**
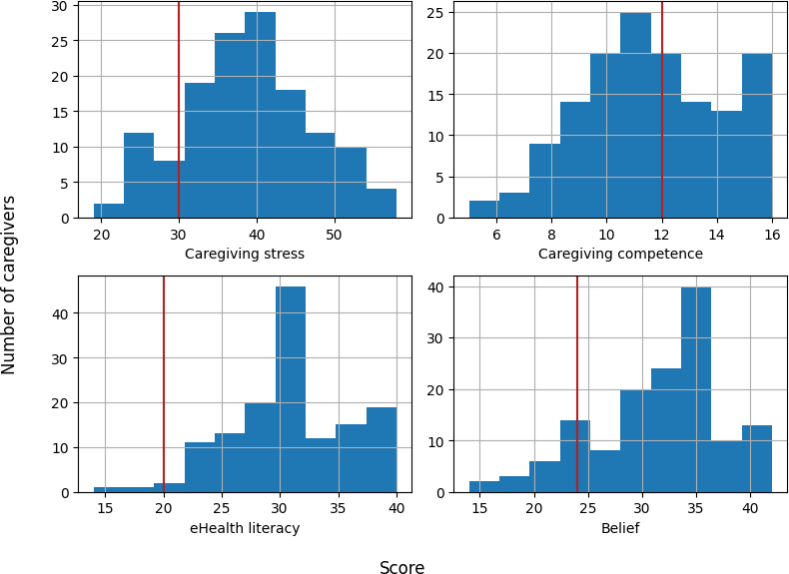
Distribution of scores for the four measures. The red vertical lines in the “Caregiving stress,” “eHealth literacy,” and “Belief” in online peer support graphs correspond to the scores when “neutral” or “undecided” was selected for all the questions in each measure. The red vertical line in the “Caregiving competence” graph corresponds to the score when the “fairly” option was selected for all the questions.

Figure 2 illustrates the distribution of the scores associated with caregiving stress, caregiving competence, eHealth literacy, and a caregiver’s belief in the value of peer support from an online environment. The Cronbach α—a measure of the internal consistency of a questionnaire or survey (the higher, the better)—of these measures was 0.86 (95% CI 0.82-0.89), 0.83 (95% CI 0.78-0.87), 0.87 (95% CI 0.84-0.90), and 0.87 (95% CI 0.83-0.89), respectively. These indicated very good internal consistency. Specifically, of the 140 caregivers, 121 (86.4%) had a stress score above 30 (the score when “neutral” was selected for all the questions; the same cutoffs were used for each measure), 67 (47.9%) had a competence score above 12, 138 (98.6%) had an eHealth literacy score above 20, and 125 (89.3%) had a belief score above 24. These results suggested that most of the caregivers were in stressful caregiving experiences, had high eHealth literacy, and believed in the value of online peer support. However, over half (73/140, 52.1%) of these caregivers were not confident in their caregiving skills.

**Figure 3.**
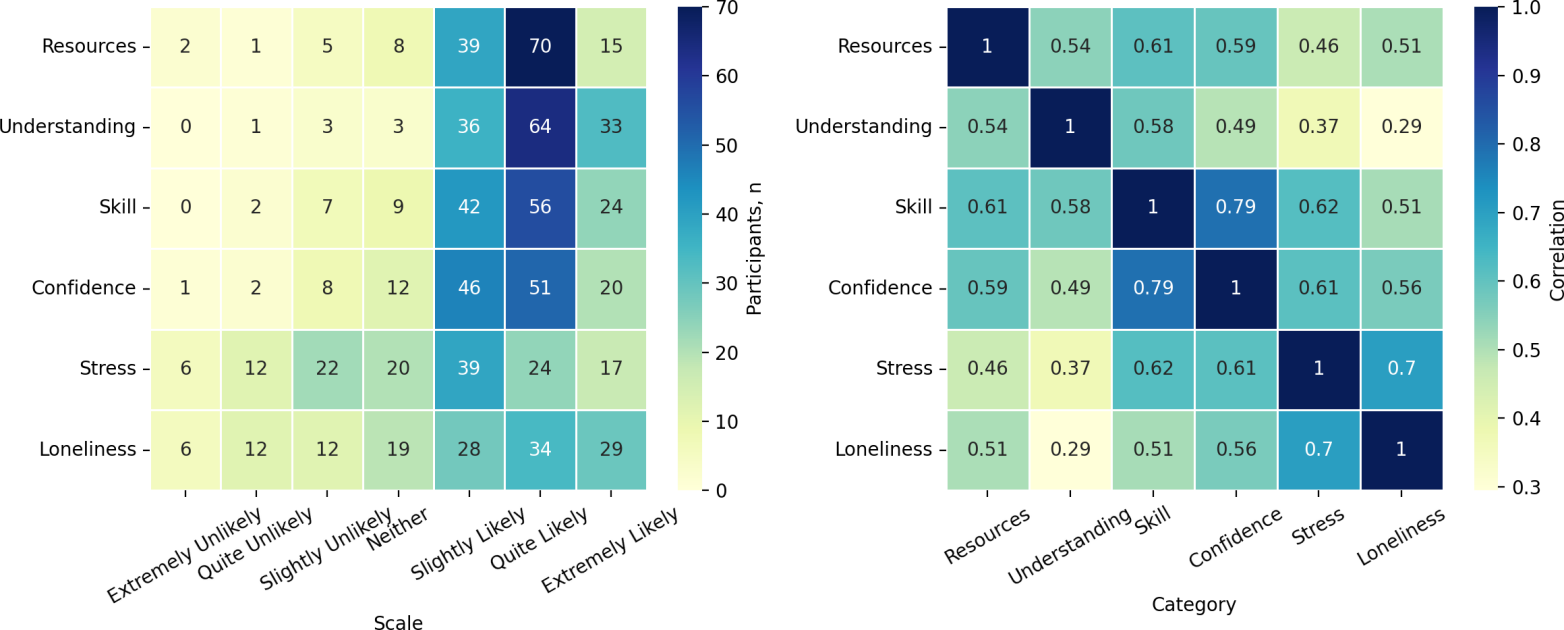
Heat maps of the number of survey participants in each belief category and scale pair (left) and the correlations in the responses (right). *Resources*: obtain the needed resources; *Understanding*: understanding patients or the disease; *Skill*: improve caregiving skills; *Confidence*: increase caregiving confidence or competence; *Stress*: reduce caregiving stress; *Loneliness*: reduce the feeling of loneliness as a caregiver.

Figure 3 provides a detailed illustration (in the form of heat maps) of the responses for each belief category and the correlations in the responses. It shows that obtaining the needed resources, increasing one’s understanding of the patient and the disease, and improving caregiving skills and confidence were the top “believed” values of online peer support. In contrast, reducing caregiving stress and the loneliness of being a caregiver was slightly more challenging. However, both categories received more “likely” responses than “unlikely” responses, with 57.1% (80/140) and 65% (91/140) of caregivers selecting “likely” responses, respectively. There were also several interesting observations made. First, reducing loneliness as a caregiver was highly correlated with reducing stress. Second, improving caregiving skills was highly correlated with increasing caregiving confidence. Third, reducing stress was correlated with improving caregiving skills and increasing caregiving confidence. Although correlation does not necessarily imply causation, these results suggested that improving caregiving skills and reducing loneliness may help to reduce caregiving stress.

### Utilization of Online Peer Support

Of the 140 survey participants, 40 (28.6%) reported never using any online community to either read online caregiving discussions or discuss caregiving issues with other online peers in the past 3 months. The regression analysis showed that only the belief score was statistically significantly associated with the utilization of online peer support (*β*: 0.11, SD 0.041; *P*=.006). All the other factors, including sociodemographics, care duration and workload, dementia stage, access to a high-speed network, eHealth literacy, stress score, and competence score, did not have statistically significant effects (all *P* values were >.10). Additionally, of the 40 non–online community caregivers, 33 (83%) had a belief score above 24—the score when “neutral” was selected for all the questions. The reasons for not using any online community to access online peer support included having no time for online activities due to the intensive caregiving workload (14/140, 10%), having a lack of searching skills (9/140, 6.4%), having belief in unreliable online information (6/140, 4.3%), and having security and privacy concerns (2/140, 1.4%). Further, 4 (2.9%) caregivers reported not wanting to spend time online after working on a computer during the daytime. Interestingly, 99 of the 100 online community caregivers reported an intention to revisit online communities in the following 3 months. The only caregiver who did not do so said that too many sad stories in online communities made her worry about her father’s future.

## Discussion

### Principal Results

When comparing with *2022 Alzheimer’s Disease Facts and Figures* (AFF) [1], we observed that our survey recruited a similar proportion of caregivers who were Hispanic caregivers (our survey: 10/140, 7.1%; AFF: 8%) and Asian caregivers (our survey: 8/140, 5.7%; AFF: 5%). However, the proportion of Black or African American caregivers we recruited was far below that reported in AFF (our survey: 5/140, 3.6%; AFF: 10%), which suggests that Asian and Hispanic caregivers are more likely to participate in research studies when compared to Black caregivers [38]. To gain greater insight into the situation, we reviewed the responses of the five caregivers who reported their race as Black. We found that two of the caregivers accessed online communities in the past 3 months, with one (age: 49 y; education level: college degree; stress score: 51) reporting this at least once per week and the other (age: 50 y; education level: more than a 4-y college degree; stress score: 19) reporting doing this when she had a caregiving question that needed an answer. Among the three remaining caregivers who reported their race as Black and did not access any online community in the past 3 months, one (age: 60 y; education level: 4-y college degree; stress score: 55) said, “I have used books and I think some information on the internet can be misinterpreted”; another one (age: 29 y; education level: some college or 2-y degree; stress score: 55) said, “In [*sic*] recently started looking for online communities”; and the third (age: 30 y; education level: some college or 2-y degree; stress score: 52) said that they were “unaware.” However, all their belief scores were above 24 and ranged from 25 to 42, which suggests that these three caregivers believed in the value of online peer support, but they may not have known how to search online communities for peer support. Notably, the caregiver who relied on books for information seeking exhibited the lowest belief score (ie, a score of 25), indicating the survey results’ reliability.

Our study shows that the most prevalent challenges faced by these informal caregivers were (1) handling the memory issues of a person living with dementia, (2) daily caregiving, (3) life balance, and (4) emotional fluctuations. Caregivers facing such challenges can, at times, receive guidance from their peers who have shared the same firsthand experiences. This suggests that online communities, such as ALZConnected, that provide an online platform for caregivers to discuss their challenges and experiences can be a valuable resource for those seeking informational or emotional peer support. This implication is further supported by our observation that a large proportion of the surveyed caregivers indicated a belief in the value of online peer support. Still, given that some caregivers did not access online environments for peer support, it is evident that online environments alone are not a comprehensive solution and that caregivers need other types of support. Moreover, there are various caregiving challenges that are more likely to be addressed by professionals rather than peers, such as clinical questions about treatment, legal issues, and financial concerns. Given that 50.7% (71/140) of the survey participants were not satisfied with their received support, it is essential to diversify the support source portfolio to assist a broad range of caregivers. As such, the use of online environments can serve as one of a collection of strategies that, in concert with one another, provide a support structure for informal caregivers.

Another main result of this study is that whether an informal dementia caregiver accessed online health communities in the past 3 months depended upon their belief in the value of peer support obtained from online health communities but not upon their sociodemographics or any other ANFHSU factors. For example, one caregiver said, “I don’t find comfort from strangers on the internet. I would love to, and I am willing to go to an in-person meeting of a support group, but I don’t have anyone to watch my mother so that I could attend,” and they exhibited a belief score of 14, which is logical based on their reason for not accessing any online communities. This further highlights the dilemma that there is limited time to attend local support groups because of intensive caregiving [15]. Another caregiver, who had a belief score of 14, indicated that their health insurance could only cover 40 days of at-home nursing support in 1 year, which made their family feel so “helpless and alone.” Despite various reasons for not using online health communities in the past 3 months, 89.3% (125/140) of respondents exhibited a belief score above 24. This suggests that online peer support was valuable to them, but an effective strategy for bridging their needs and the desired online peer support is needed. Finally, the correlation of the survey responses to the six value aspects implied that an intervention designed around peer learning can effectively enhance a caregiver’s caregiving competence and reduce feelings of loneliness.

### Limitations

There are, however, several limitations to this study that can serve as a basis for future research. First, since we distributed our survey link in the ALZConnected online community and the Alzheimer’s Association’s website, our results may be biased toward online community users. A less biased approach may be designed to collect data that reflect the perceptions and utilization of online peer support in the dementia caregiver population. Making online peer support beneficial to noninternet caregivers is equally essential, but determining how to address the internet access issue is a priority and is beyond the scope of this study. Second, only a small percentage of the participants were caregivers who reported their race as Black. Increasing participation in this group would increase the understanding of their perceptions and utilization of online peer support. Third, the analysis relied on 140 completed responses, indicating limited statistical power. Although statistically significant findings emerged, larger sample sizes are necessary for a more precise examination of this research. Fourth, in an open-ended question, we inquired about the types of offline support caregivers received to address their caregiving challenges. Some caregivers mentioned online peer support in their responses, leading to misalignment between their answers and the original research design, particularly regarding satisfaction with offline support. Future investigations should clarify this question or convert this question to a multiple-choice version. Finally, it is essential to study how to help caregivers without time or sufficient online information–searching skills screen the online caregiving discussions they need.

### Conclusions

This study reported on an online survey about the perceptions and utilization of online peer support among informal dementia caregivers. Belief in the value of online peer support was statistically significantly associated with accessing online communities (*P*=.006). Moreover, there were a number of caregivers who were not using online peer support but held belief in the value of such support. As such, there is clearly an opportunity to build tools that help caregivers who are existing online community users and caregivers who intend to seek online information find reliable, matched online peer support.

## Abbreviations

AFF: *2022 Alzheimer’s Disease Facts and Figures*
ANFHSU: Andersen and Newman Framework of Health Services Utilization
CARERS: Coaching, Advocacy, Respite, Education, Relationship, and Simulation
eHEALS: eHealth Literacy Scale
REDCap: Research Electronic Data Capture
